# Isocitrate dehydrogenase mutation in *Vibrio anguillarum* results in virulence attenuation and immunoprotection in rainbow trout (*Oncorhynchus mykiss)*

**DOI:** 10.1186/s12866-017-1124-1

**Published:** 2017-11-14

**Authors:** Xiangyu Mou, Edward J. Spinard, Shelby L. Hillman, David R. Nelson

**Affiliations:** 1 Department of Cell and Molecular Biology, University of Rhode Island, Kingston, RI 02881 USA; 2 Present Address: Division of Infectious Diseases, Massachusetts General Hospital, 65 Landsdowne St, Cambridge, MA 02139 USA

**Keywords:** *Vibrio anguillarum*, TCA cycle, Vibriosis, Isocitrate dehydrogenase, Virulence, Hemolysin

## Abstract

**Background:**

*Vibrio anguillarum* is an extracellular bacterial pathogen that is a causative agent of vibriosis in finfish and crustaceans with mortality rates ranging from 30% to 100%. Mutations in central metabolism (glycolysis and the TCA cycle) of intracellular pathogens often result in attenuated virulence due to depletion of required metabolic intermediates; however, it was not known whether mutations in central metabolism would affect virulence in an extracellular pathogen such as *V. anguillarum*.

**Results:**

Seven central metabolism mutants were created and characterized with regard to growth in minimal and complex media, expression of virulence genes, and virulence in juvenile rainbow trout (*Oncorhynchus mykiss*). Only the isocitrate dehydrogenase (*icd*) mutant was attenuated in virulence against rainbow trout challenged by either intraperitoneal injection or immersion. Further, the *icd* mutant was shown to be immunoprotective against wild type *V. anguillarum* infection. There was no significant decrease in the expression of the three hemolysin genes detected by qRT-PCR. Additionally, only the *icd* mutant exhibited a significantly decreased growth yield in complex media. Growth yield was directly related to the abundance of glutamate. A strain with a restored wild type *icd* gene was created and shown to restore growth to a wild type cell density in complex media and pathogenicity in rainbow trout.

**Conclusions:**

The data strongly suggest that a decreased growth yield, resulting from the inability to synthesize α-ketoglutarate, caused the attenuation despite normal levels of expression of virulence genes. Therefore, the ability of an extracellular pathogen to cause disease is dependent upon the availability of host-supplied nutrients for growth. Additionally, a live vaccine strain could be created from an *icd* deletion strain.

**Electronic supplementary material:**

The online version of this article (10.1186/s12866-017-1124-1) contains supplementary material, which is available to authorized users.

## Background

The aquaculture industry now produces half of all fish intended for human consumption and employs millions of people worldwide [1]. Although the first value sale of harvested fish has increased by 267% between 2004 and 2014 to over US$160 billion, infectious diseases, especially those caused by *Vibrio* spp. including *Vibrio anguillarum*, still represent a major impediment to the production of fish [1]*. V. anguillarum* causes diseases in crustaceans and bivalves, and is the leading causative agent of vibriosis in finfish including salmon, rainbow trout, turbot, sea bass, sea bream, cod, eel, and ayu [2]. Infections by this bacterial species have resulted in severe economic losses to aquaculture industries worldwide [3].

*V. anguillarum* is an extracellular pathogen that invades its host fish through the intestine, skin or gills [4, 5]. Systemic infection by *V. anguillarum* usually causes fish to die within 1–4 days [6–9]. Chemotactic motility and the metalloprotease EmpA have been shown to be important virulence factors during the invasion stage while the siderophore anguibactin, flagellin subunits and lipopolysaccharides were shown to be important for persistence in the host during the post-invasion stage [2, 10]. Three secreted proteins that are cytotoxic against epithelial cells and erythrocytes have been characterized in *V. anguillarum*: the HlyA homolog Vah1, the phospholipase Plp, and the MARTX toxin RtxA [7, 9, 11]. Mutations in *vah1* and/or *plp* resulted in slight attenuation against juvenile Atlantic salmon (*Salmo salar*); however, *rtxA* mutants were avirulent [7, 9, 11]. Additionally, a *V. anguillarum* mutant that lacks H-NS, a global transcriptional regulator that represses the transcription of *vah1*, *plp*, and *rtxA*, showed attenuation in virulence when injected intraperitoneally, suggesting that proper coordination of gene expression is an important factor during the post-invasion stage [8].

Since the 1980s, several bacterial species that are auxotrophic for aromatic compounds have been shown to be avirulent [12–16]. More recently, mutants that are hypothesized to experience growth defects in the nutrient limited environment inside a phagocyte have been characterized. In *Salmonella enterica,* an intracellular bacterial pathogen, some tricarboxylic acid (TCA) cycle mutant strains were avirulent and immunoprotective for subsequent wild-type *S. enterica* infection [17–21]. A functional fructose repressor (Cra) was also required for *S. enterica* infection [22]. Similar results have been observed for central metabolism mutants in other intracellular pathogens such as uropathogenic *Escherichia coli* (UPEC), *Mycobacterium tuberculosis*, and the facultative intracellular fish pathogen *Edwardsiella ictaluri* [23–27]. These observations demonstrate that central metabolism is important for pathogenesis by intracellular pathogens.

Accordingly, we hypothesized that mutations in central metabolism could interrupt the infection process of *V. anguillarum* in juvenile rainbow trout *(Oncorhynchus mykiss)*. In this study, we identified and created six TCA cycle mutant strains plus one fructose metabolism mutant strain, and tested their virulence against juvenile rainbow trout using two infection methods, intraperitoneal (IP) injection and immersion. Further, the expression of each of the three hemolysin genes (*vah1*, *plp*, and *rtxA*) was examined to determine whether attenuation resulted from decreased virulence factor expression in these mutants. The growth rates and yield of each mutant strain in complex media were also determined. We specifically characterized the growth defect of the attenuated *icd* mutant. We also created, tested, and compared a restored wild type *icd* strain for virulence and growth to both the wild type and the *icd* mutant.

## Methods

### Bacterial strains, plasmids and growth conditions

*V. anguillarum* strains (Table 1) were routinely grown in Lysogeny broth containing 2% NaCl (LB20) [28] or Marine Minimum Median (3M) + 0.15% glucose [29], supplemented with the appropriate antibiotic, in a shaking water bath at 27 °C. *E. coli* strains (Table 1) were routinely grown in Lysogeny broth containing 1% NaCl (LB10) supplemented with the appropriate antibiotic, in a shaking water bath at 37 °C. Antibiotics were used at the following concentrations: streptomycin, 200 μg/ml (Sm^200^); chloramphenicol, 20 μg/ml (Cm^20^) for *E. coli* and 5 μg/ml (Cm^5^) for *V. anguillarum*; kanamycin, 50 μg/ml (Km^50^) for *E. coli* and 80 μg/ml (Km^80^) for *V. anguillarum.*

**Table 1:** Bacterial strains and plasmids used in this study

Strain or plasmid	Description	Reference
*V. anguillarum* strains		
M93Sm	Spontaneous Sm^r^ mutant of M93 (serotype O2a)	[47]
XM420	Sm^r^ Cm^r^; *icd* insertional mutant	This study
ES422	Sm^r^; Restored *icd* strain	This study
XM440	Sm^r^ Cm^r^; *sucA* insertional mutant	This study
XM450	Sm^r^ Cm^r^; *sucC* insertional mutant	This study
XM460	Sm^r^ Cm^r^; *sdhC* insertional mutant	This study
XM470	Sm^r^ Cm^r^; *fumA* insertional mutant	This study
XM410	Sm^r^ Cm^r^; *mdh* insertional mutant	This study
XM430	Sm^r^ Cm^r^; *cra* insertional mutant	This study
*E. coli* strains		
SM10	*thi thr leu tonA lacY supE recA* RP4–2-Tc::Mu::Km (λ *pir*)	[48]
S100	Km^r^; Sm10 containing plasmid pNQ705–1	[49]
Q420	Km^r^ Cm^r^; Sm10 containing plasmid pNQ705-*icd*	This study
Q440	Km^r^ Cm^r^; Sm10 containing plasmid pNQ705-*sucA*	This study
Q450	Km^r^ Cm^r^; Sm10 containing plasmid pNQ705-*sucC*	This study
Q460	Km^r^ Cm^r^; Sm10 containing plasmid pNQ705-*sdhC*	This study
Q470	Km^r^ Cm^r^; Sm10 containing plasmid pNQ705-*fumA*	This study
Q410	Km^r^ Cm^r^; Sm10 containing plasmid pNQ705-*mdh*	This study
Q430	Km^r^ Cm^r^; Sm10 containing plasmid pNQ705-*cra*	This study
Plasmid		
pNQ705–1	Cm^r^; suicide vector with R6K origin	[49]
pNQ705-*icd*	Cm^r^; For *icd* insertional mutant	This study
pNQ705-*sucA*	Cm^r^; For *sucA* insertional mutant	This study
pNQ705-*sucC*	Cm^r^; For *sucC* insertional mutant	This study
pNQ705*-sdhC*	Cm^r^; For *sdhC* insertional mutant	This study
pNQ705-*fumA*	Cm^r^; For *fumA* insertional mutant	This study
pNQ705*-mdh*	Cm^r^; For *mdh* insertional mutant	This study
pNQ705-*cra*	Cm^r^; For *cra* insertional mutant	This study

### Identification of genes in *V. anguillarum*

*V. anguillarum* M93Sm draft genome (accession number NOWD00000000) was annotated by the RAST (Rapid Annotation using Subsystem Technology) service (http://rast.nmpdr.org/rast.cgi) using the default settings [30]. The following annotated genomes were downloaded from NCBI: *V. anguillarum* 775 (accession numbers: NC_015633.1 and NC_015637.1), 96F (accession number: NZ_AEZA00000000.1), M3 (accession numbers: NC_022223.1, NC_022224.1 and NC_022225.1), NB10 (accession numbers: NZ_LK021130.1, NZ_LK021129.1 and NZ_LK021128.1), RV22 (accession number: AEZB00000000.1) and 90–11-286 (accession numbers: NZ_CP011460.1 and NZ_CP011461.1).

### Insertional mutagenesis

Insertional mutations were made by using a modification of the procedure described by Milton et al. [31]. Briefly, primers (Table 2) were designed based on the target gene sequence of M93Sm. An internal 200–300 bp DNA fragment of the first third of the target gene was PCR amplified and ligated into the suicide vector pNQ705–1 (Table 1) after digestion with SacI and XbaI. The ligation mixture was introduced into *E. coli* SM10 by electroporation using a BioRad Gene Pulser II (BioRad, Hercules, CA). Transformants were selected on LB10 Cm^20^ agar plates. The construction of the recombinant pNQ705 was confirmed by both PCR amplification and restriction enzyme analysis. The mobilizable suicide vector was transferred from *E. coli* SM10 into *V. anguillarum* by conjugation [32]. Transconjugants were selected by utilizing the chloramphenicol resistance gene located on the suicide plasmid. The incorporation of the recombinant pNQ705 was confirmed by PCR amplification.

**Table 2:** Primers used in this study

Primer	Sequence (5′ to 3′, underlined sequences are designed restriction sites)	Description	Reference
pr31	GGTGAGCTCTATTCTTTATTGCCGATTATC	For *icd* insertional mutant, forward, *Sac*I	This study
pr32	AAATCTAGAGTAAGTCGCTTTAATCGCTTC	For *icd* insertional mutant, reverse, *Xba*I	This study
pr50	AAAGAGCTCGTGATCCAGATGTCGATGCTA	For *sucA* insertional mutant, forward, *Sac*I	This study
pr51	GGTTCTAGAGTTCAGTGTCGATAATGTGCA	For *sucA* insertional mutant, reverse, *Xba*I	This study
pr52	AAAGAGCTCGGTCGGATTAGTACAGCGAAG	For *sucC* insertional mutant, forward, *Sac*I	This study
pr53	GGTTCTAGACTTTTTCAATTTCCACGCCGC	For *sucC* insertional mutant, reverse, *Xba*I	This study
pr54	AAAGAGCTCATGTTCGTTGCGGTCGGAATT	For *sdhC* insertional mutant, forward, *Sac*I	This study
pr55	GGTTCTAGATCCAACTCTTCAAAGTGGCCC	For *sdhC* insertional mutant, reverse, *Xba*I	This study
pr56	GGTGAGCTCTCCTTGCACCATATTGATATG	For *fumA* insertional mutant, forward, *Sac*I	This study
pr57	GGGTCTAGAAGGCTTATCATCGAGAAGAGAG	For *fumA* insertional mutant, reverse, *Xba*I	This study
pr29	GGTGAGCTCATGCCAGCGTTAACATTAAAC	For *mdh* insertional mutant, forward, *Sac*I	This study
pr30	AAATCTAGAGCTGTATGACATCGCACCGGT	For *mdh* insertional mutant, reverse, *Xba*I	This study
pr33	AAAGAGCTCGCGGCGTGAGACTAAGGCATC	For *cra* insertional mutant, forward, *Sac*I	This study
pr34	AAATCTAGACAATGGCAAAGCGCAGAAGTA	For *cra* insertional mutant, reverse, *Xba*I	This study
vah1 F RT	GTTTGGTATGGAACACCGCTCAAG	For *vah1* qRT-PCR, forward	This study
vah1 R RT	GGCTCAACCTCTCCTTGTAACCAA	For *vah1* qRT-PCR, reverse	This study
plp F RT	CAGACGACCACCAGTAACCACTAA	For *plp* qRT-PCR, forward	[8]
plp R RT	GCAATCATGATGACCCAGCAACAG	For *plp* qRT-PCR, reverse	[8]
Pm111	GGAAATTATTCCGCCGACGATGGA	For *rtxA* qRT-PCR, forward	[7]
Pm112	GCCGATACCGTATCGTTACCTGAA	For *rtxA* qRT-PCR, reverse	[7]

### Fish infection experiments

Various *V. anguillarum* strains were tested for virulence against rainbow trout *(O. mykiss)* by intraperitoneal (IP) injection or immersion. Briefly, *V. anguillarum* cells grown for 19 h at 27 °C in LB20 supplemented with the appropriate antibiotics were harvested by centrifugation (9000×*g*, 5 min, 4 °C), washed twice in nine salts solution (NSS), and resuspended in NSS [33, 34]. Aliquots (100 μl) of the *V. anguillarum* NSS suspension were used to determine the OD_600_. The *V. anguillarum* NSS suspension was prepared to the desired specific cell density according to the conversion equation as determined by experimentation (data not shown): Cell density (10^8^ CFU/ml) = 44.905 × OD_600_. The actual cell density of the suspension was confirmed by dilution and viable plate count. All fish were examined and determined to be disease and injury free prior to the start of each experiment. For IP injection, fish were anesthetized by tricaine methanesulfonate (Western Chemical, Ferndale, WA), (100 mg/l for induction and 52.5 mg/l for maintenance). *V. anguillarum* strains were IP injected into fish that were between 15 and 25 cm long in a 100 μl NSS vehicle at a dose of either 2 × 10^5^ or 4 × 10^5^ CFU/fish, or with NSS only as a negative control. For immersion, 10 ml of *V. anguillarum* suspended in NSS, or 10 ml of NSS only as a negative control was added to a bucket filled with 10 L of water supplemented with 1.5% NaCl that was maintained at 18 ± 1 °C. Fish that were between 15 and 25 cm long were added and immersed for 1 h. For both methods, fish inoculated with different bacterial strains were maintained in separate 10-gal (38 L) tanks to prevent possible cross-contamination with constant water flow (200 ml/min) at 18 ± 1 °C. Death due to vibriosis was determined by the observation of gross clinical symptoms and confirmed by the recovery and isolation of *V. anguillarum* cells resistant to the appropriate antibiotics from the spleen or head kidney of dead fish. Observations were made for 8–14 days. All fish used in this research project were obtained from the URI East Farm Aquaculture Center. All fish infection protocols were approved by the URI IACUC. (IACUC Protocol AN06–08-002).

### RNA isolation

Exponential phase cells (~0.5 × 10^8^ CFU/ml) of various *V. anguillarum* strains were treated with RNAprotect Bacteria Reagent (QIAGEN), following the manufacturer’s instructions. Total RNA was isolated using the RNeasy kit and QIAcube (QIAGEN) following the instructions of the manufacturer. All purified RNA samples were quantified spectrophotometrically by measuring absorption at 260 nm and 280 nm using a NanoDrop ND-1000 spectrophotometer (Thermo Fisher Scientific) and overall quality was assessed by gel electrophoresis. Samples were stored at −75 °C for future use.

### Real-time quantitative RT-PCR (qRT-PCR)

qRT-PCR was used to quantify various mRNAs using an LightCycler® 480 Real-Time PCR System (Hoffmann-La Roche Inc.) and the Brilliant II SYBR Green Single-Step QRT-PCR Master Mix (Agilent Technologies), with 10 ng of total RNA in 20 μl reaction mixtures. The thermal profile was 50 °C for 30 min, 95 °C for 15 min, and then 40 cycles of 95 °C for 30 s and 55 °C for 30 s. Fluorescence was measured at the end of the 55 °C stage of each cycle. Samples were run in triplicate along with the no-reverse-transcriptase control and the no-template control. All experiments were repeated at least twice.

### Growth experiments

To cultivate bacteria for growth experiments, *V. anguillarum* cells grown overnight at 27 °C in LB20 supplemented with the appropriate antibiotics were harvested by centrifugation (9000×*g*, 2 min), washed twice and resuspended in in NSS. A 200 μl aliquot of the *V. anguillarum* NSS suspension was transferred into a 96-well plate with a clear flat bottom and the optical density at 600 nm (OD_600_) was read by a VersaMax™ Absorbance Microplate Reader (Molecular Devices). The *V. anguillarum* NSS suspension was prepared to an OD_600_ of 0.420 (~4 × 10^7^ CFU/ml) and diluted 1:100 into fresh media. Growth was monitored either by measurement of the OD_600_ or by serial dilution and plate counts.

### Resolving the merodiploid in the *icd* mutant

*V. anguillarum icd* mutant cells grown in LB20 supplemented with appropriate antibiotics for 19 h at 27 °C were harvested by centrifugation (9000×*g*, 2 min), washed three times in NSS, and resuspended in NSS. Cell suspensions (100 μl) were spread onto Marine Minimum Median (3 M) + 0.15% glucose agar. Well-isolated colonies were picked and subsequently streak purified onto a new 3 M + 0.15% glucose agar. Isolated colonies were then transferred to LB20Cm^5^ agar to screen for chloramphenicol sensitivity. Resolution of the merodiploid was confirmed by PCR amplification.

### Statistical analysis

A Kaplan-Meier survival analysis with log rank significance test was performed on the survival curves in the fish infection experiment. Student’s T-tests assuming unequal variances were used for experiments containing two data groups. One-way ANOVA with Tukey post hoc test was performed for all other experiments. *P* values of <0.05 were considered statistically significant.

## Results

### Identification and mutant construction of TCA cycle genes in *V. anguillarum*

**Fig. 1:**
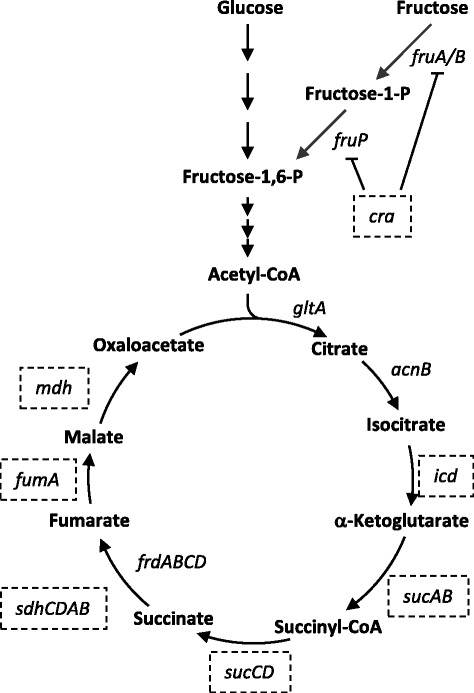
Embden-Meyerhoff-Parnas Pathway, TCA cycle, and metabolism of fructose. The arrows indicate the physiological directions of the reactions. The gene symbols of the enzyme for each reaction are listed beside the reaction. Boxed genes indicate the genes that were mutated in this study (Table 1)

In order to identify gene targets for mutagenesis the published genomes of *V. anguillarum* strains 775, 96F, M3, NB10, RV22, 90-11-286 and the *V. anguillarum* M93Sm draft genome (accession number NOWD00000000) annotated by RAST were examined and found to have the following TCA cycle genes/operons: *gltA*, *acnB, icd, sucAB, sucCD, sdhCDAB, frdABCD fumA,* and *mdh* (Fig. 1 and Table 3). While this set of genes allows for a fully functional TCA cycle, none of the strains have a *fumC* gene, which encodes the aerobic fumarate class II hydratase. All strains also lack the anaerobic fumarate hydratase (*fumB*) gene. Additionally, all strains possessed *cra*, which encodes the repressor of fructose metabolism in *S. enterica* [22]. The *V. anguillarum* M93Sm sequences for the *icd*, *sucA, sucC, sdhC, fumA, mdh*, and *cra* genes were used to create insertional mutations in *V. anguillarum* M93Sm. The seven mutant strains and the one restored strain listed in Table 1 were constructed using the primers listed in Table 2 as described in the Methods.

**Table 3:** Metabolism genes examined in this study

Gene or operon	Product	Present in sequenced *V. anguillarum* strains ^a^
*gltA*	Type II citrate synthase	Yes
*acnB*	Aconitate hydratase B	Yes
*icd*	Isocitrate dehydrogenase	Yes
*sucAB*	2-oxoglutarate dehydrogenase (E1 component, E2 component)	Yes
*sucCD*	Succinyl-CoA synthetase (beta subunit, alpha subunit)	Yes
*sdhCDAB*	Succinate dehydrogenase (cytochrome b556 subunit, membrane anchor subunit, flavoprotein subunit, iron-sulfur protein)	Yes
*frdABCD*	Fumarate reductase (flavoprotein subunit, iron-sulfur subunit, anchor subunit, anchor subunit)	Yes
*fumAC*	Aerobic fumarate hydratase (class I, class II)	*fumA*: Yes*; fumC*: not found
*fumB*	Anaerobic fumarate hydratase (class I)	Not found
*mdh*	Malate dehydrogenase	Yes
*cra*	Fructose repressor protein	Yes

^a^ *V. anguillarum* strains: M93Sm, 775, 96F, M3, NB10, RV22, 90-11-286

### *icd* mutant is highly attenuated for virulence against rainbow trout

**Fig. 2:**
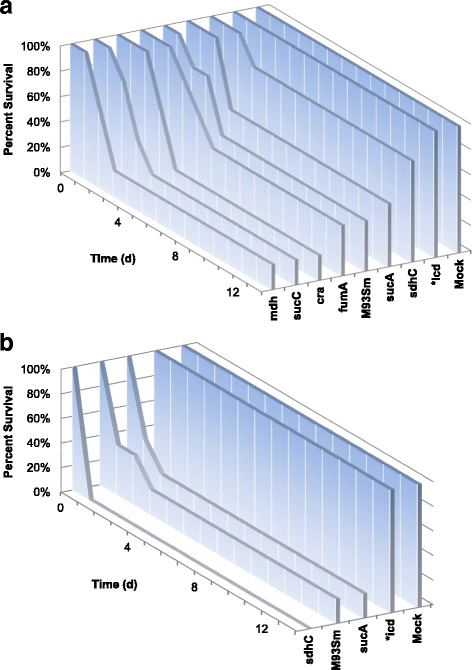
Percent survival of rainbow trout IP injected with *V. anguillarum* wild type (M93Sm) and various mutant strains at a dosage of **a** 2 × 10^5^ CFU/fish and **b** 4 × 10^5^ CFU/fish. Negative control groups of fish (Mock) were injected with sterile NSS. Five fish were used for each treatment. (One fish treated with the *sucA* mutant died, but not from vibriosis and no *V. anguillarum* were recovered, so only four fish were counted). *Statistically significant difference compared to M93Sm (*p* < 0.05)

The virulence of the seven *V. anguillarum* metabolism mutants were tested on rainbow trout and compared to wild type M93Sm in order to determine if mutations in metabolism could affect pathogenesis. Groups of five fish were infected by IP-injection (as described in the Methods) with either the wild type (M93Sm), *icd* mutant (XM420), *sucA* mutant (XM440)*, sucC* mutant (XM450)*, sdhC* mutant (XM460)*, fumA* mutant (XM470)*, mdh* mutant (XM410) or *cra* mutant (XM430) in NSS at a dosage of ~2 × 10^5^ CFU per fish. Injection with NSS only served as a negative control (Mock). During the 14-day observation window, 40% of M93Sm infected fish survived. Fish infected with the *sucA* mutant*, sdhC* mutant or *icd* mutant had a higher survival percentage than M93Sm (50% for *sucA* mutant, 80% for *sdhC* mutant, and 100% for *icd* mutant); however, only the difference between the *icd* mutant and M93Sm was statistically significant (*p* = 0.037) (Fig. 2a). The experiment was repeated using a two-fold higher dose (~4 × 10^5^ CFU per fish) of M93Sm and the three mutant strains (*icd* mutant, *sucA* mutant and *sdhC* mutant) that exhibited attenuated virulence in the previous experiment. At this dose, only 20% of M93Sm-infected fish survived. Only the *icd* mutant-infected fish had a statically significant higher survival percentage (100%) compared to M93Sm (*p* = 0.0153) (Fig. 2b). The data indicate the *icd* mutant is avirulent in these experimental conditions.

**Fig. 3:**
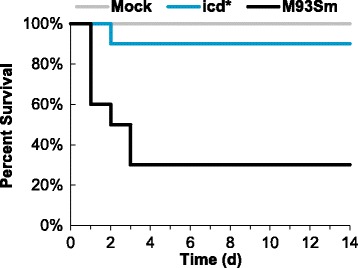
Percent survival of rainbow trout infected by immersion with *V. anguillarum* strains M93Sm (wild type) or XM420 (*icd*) at a dose of 4 × 10^6^ CFU/ml. A negative control group of fish (Mock) was immersed in sterile NSS. Ten fish were used for each treatment. *Statistically significant difference compared to M93Sm (*p* < 0.05)

Further, we tested the virulence of M93Sm and the *icd* mutant by another infection route. Groups of 10 fish were infected by immersion as described in the Methods with M93Sm or *icd* mutant in 1.5% salt solution at a dose of ~4 × 10^6^ CFU/ml, or just immersed in a 1.5% salt solution without *V. anguillarum* as a negative control (Mock). During the 14-day observation window, there was a statistically significant difference (*p* = 0.007) between the survival of M93Sm infected fish (30%) and *icd* mutant infected fish (90%) (Fig. 3). Taken together, the IP infection data and the immersion infection data demonstrate that the *icd* mutant is highly attenuated for infection in rainbow trout.

### Pre-treatment by immersion with the *icd* mutant protected rainbow trout from the subsequent challenge of *V. anguillarum* M93Sm

**Fig. 4:**
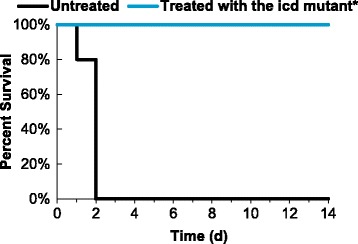
Percent survival of immersion vaccinated rainbow trout. Rainbow trout were sham vaccinated with NSS (labeled as “untreated”) or immersed vaccinated with the *icd* mutant (Labeled as “treated with icd”) and challenged with wild type *V. anguillarum* M93Sm (4 × 10^6^ CFU/ml). Five fish were used for each treatment. *Statistically significant difference compared to M93Sm (*p* < 0.05)

Fish previously challenged by immersion with the *icd* mutant were subsequently challenged with the wild type M93Sm strain to test if the *icd* mutant was immunogenic. Six weeks after the initial infection, a group of five fish that survived the initial infection with the *icd* mutant (labeled as “treated with the *icd* mutant” in Fig. 4) and a group of five “untreated” fish were infected via immersion with M93Sm at a dose of ~4 × 10^6^ CFU/ml and were observed for 14 days. By day 2 all fish in the untreated group died. All fish in the group treated with the *icd* mutant survived the 14-day observation period. The difference between the two experimental groups was statistically significant (*p* = 0.008). The results indicate that the *icd* mutant is immunogenic and protective against wild type infection when administered by immersion.

### All mutants exhibited either same or higher expression levels of the three hemolysin genes compared to wild type

**Fig. 5:**
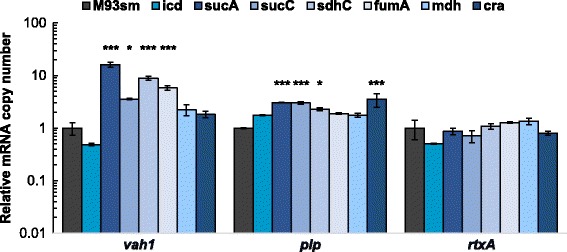
Relative expression of *vah1*, *plp*, *rtxA* determined by qRT-PCR analysis of *V. anguillarum* wild-type (M93Sm) and various TCA mutants during logarithmic (Log)-phase growth. The data presented are representative of two independent experiments. Each value is the average for three replicates. Between marked strains and M93Sm: * *p* < 0.05 and *** *p* < 0.001. Error bars represent 1 standard deviation

Vah1, RtxA, and Plp are the three hemolysins found in M93Sm and are responsible for the hemolytic/cytolytic activity against fish erythrocytes, leukocyte and epithelial cells [7, 9, 11] and unpublished data]. We tested the expression of *vah1*, *rtxA* and *plp* during exponential phase to determine whether mutations in metabolism could affect the expression of these hemolysin genes. Data indicate that in all mutants except the *icd* mutant, expression of *vah1* and *plp* were up regulated by 1.49–16.15-fold compared to M93Sm with most of the changes being significant (Fig. 5). In the *icd* mutant, expression of *plp* was up regulated by 1.76-fold while the expression of *vah1* was slightly decreased (to 49% of WT), neither of which was a significant change from M93Sm (Fig. 5). Plp is the most efficient hemolysin against fish erythrocytes [11]. TCA cycle mutants with an increased expression of *plp* also demonstrated an increased zone of hemolysis on 5% fish blood agar plates (Additional file 1: Figure S1). There was no change in the zone of hemolysis for the *icd* mutant. Expression of *rtxA* in all mutants was not significantly different from M93Sm (Fig. 5). Taken together, all metabolism mutants have the same or higher expression levels of hemolysin genes compared to the wild type.

### *icd* mutant exhibited significant lower cell density limit than wild type in two forms of rich media

**Fig. 6:**
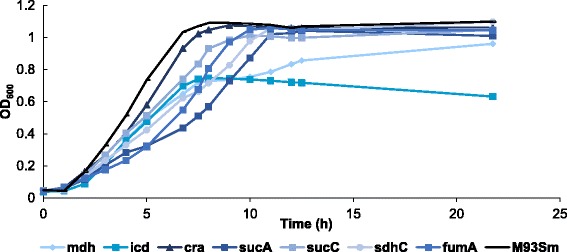
Growth curves of various *V. anguillarum* strains grown in LB20 at 27 °C with shaking (200 rpm). At various time points after inoculation samples were taken for determination of optical density at 600 nm (OD_600_). The data are from one representative experiment

Figure 6 shows the typical growth curves for the wild type *V. anguillarum* M93Sm and the seven metabolism mutants in LB20 broth. In these growth conditions, M93Sm, the *icd* mutant, and the *cra* mutant exhibited classic bacterial growth curves with a lag phase, an exponential phase and a stationary phase. The *sucA, sucC, sdhC, fumA* and *mdh* mutants all exhibited a two-stage growth curve, with each stage consisting of a lag phase and an exponential phase. The exponential phase in the first growth stage was named exponential phase I and the exponential phase in the second growth stage was named exponential phase II. The generation times of the exponential phases of all mutants were longer than for M93Sm (Table 4). The final cell density (measured by OD_600_) of the *icd* mutant after 23 h was the lowest among all strains. Similarly, after 24 h of growth in LB20 the final cell density (CFU/ml) of the *icd* mutant was 47% that of M93Sm (Table 5) and the difference is significant (*p* = 0.011). M93Sm and the *icd* mutant were grown in NSS supplemented with 200 μg protein/ml of fish gastrointestinal mucus (NSSM) to better replicate conditions within a host. After 24 h of growth in NSSM the final cell density of the *icd* mutant was only ~31% of that for M93Sm (Table 5) and the difference is significant (*p* = 0.007).

**Table 4:** Generation times of various *V. anguillarum* strains grown in LB20^a^

Strain	Exponential Phase I (Minutes)	Exponential Phase II (Minutes)
M93Sm	44.00	NA
*icd*	54.95	NA
*sucA*	64.32	98.52
*sucC*	52.42	99.57
*sdhC*	61.19	101.70
*fumA*	73.55	89.38
*mdh*	67.11	115.28
*cra*	58.59	NA

*NA* not applicable

^a^Values calculated from data presented in Fig. 6 during exponential growth

**Table 5:** Final cell density (CFU/ml) of various *V. anguillarum* cultures grown for 24 h

Strain	CFU/ml in LB20	CFU/ml in NSSM (200 μg/ml)
M93Sm	3.4 × 10^9^ (±0.3 × 10^9^)	4.2 × 10^9^ (±0.7 × 10^9^)
*icd*	1.6 × 10^9^ (±0.02 × 10^9^)*	1.3 × 10^9^(±0.3 × 10^9^)*

*Statistically significant difference compared to M93Sm (*p* < 0.05)

### Growth in LB20 supplemented with 118 mM glutamate restores growth of the *icd* mutant to wild type levels

**Fig. 7:**
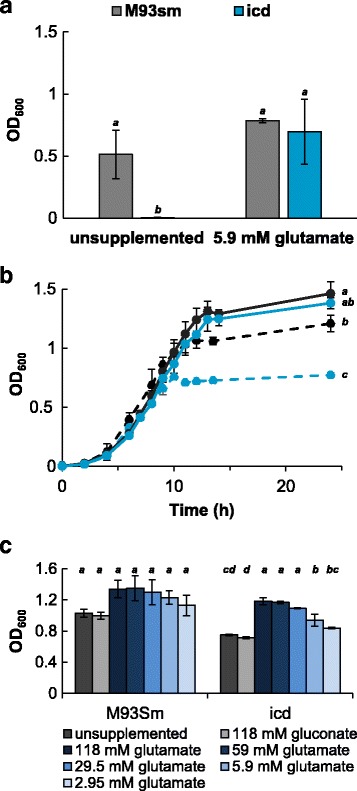
Growth of *V. anguillarum* WT (M93Sm) and the *icd* mutant under various conditions. **a** Final cell densities (OD_600_) of *V. anguillarum* strains after 24 h of growth in 3M plus 0.15% glucose supplemented with or without 5.9 mM glutamate. **b** Growth curves of *V. anguillarum* M93Sm (black) and the *icd* mutant (blue) in LB20 (dashed lines) or LB20 supplemented with 118 mM glutamate (solid lines). Statistical analysis was based on data at 24 h. **c** Final cell densities (OD_600_) of *V. anguillarum* M93Sm and *icd* mutant strains grown in LB20 supplemented with decreasing amounts of glutamate. In each experiment cells grown overnight in LB20 were washed in NSS and used to inoculate the appropriate media. Cultures were incubated at 27 °C in a shaking water bath (200 rpm) and at various time points after inoculation samples were taken for determination of optical density at 600 nm (OD_600_). Different letters indicate statistical significance among groups (*p* < 0.05). Error bars represent 1 standard deviation

The *icd* mutant is unable convert isocitrate into α-ketoglutarate, the immediate precursor of glutamate. Consequently, the *icd* mutant was only able to grow in 3 M + 0.15% glucose with the addition of glutamate (Fig. 7a). Glutamate was added to LB20 to determine if the *icd* mutant final cell density would increase. Figure 7b shows the typical growth curves of M93Sm and the *icd* mutant in LB20 with (solid lines) and without (dashed lines) the addition of 118 mM of glutamate. After 24 h, M93Sm and the *icd* mutant grew to similar final cell densities when LB20 was supplemented with 118 mM glutamate. Additionally, Fig. 7c demonstrate that decreasing the amount of glutamate (from 118 mM to 2.95 mM) added to LB20 decreases the final cell density of the *icd* mutant, but not M93Sm, after 24 h of growth. The final cell density of the *icd* mutant was not restored to a wild type level when LB20 was supplemented with glucose, succinate (Additional file 2: Figure S2) or gluconate (Fig. 7c).

### Resolving the merodiploid in the *icd* mutant restores growth and pathogenicity

**Fig. 8:**
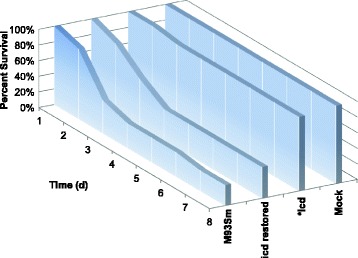
Percent survival of rainbow trout immersed with various *V. anguillarum* strains at a dosage of 4 × 10^6^ to 7 × 10^6^ CFU/ml. Five fish were used for the uninfected (mock) group. Fifteen fish were treated with the restored *icd* strain. Nineteen fish were treated with M93Sm and twenty fish were treated with the *icd* mutant. *Statistically significant difference compared to M93Sm (*p* < 0.01)

A revertant to the wild type *icd* gene was selected to demonstrate that the *icd* mutant (XM420, a merodiploid with an insertion in the *icd* gene) contained no additional mutations that could be causing the loss of pathogenicity and decreased cell density. Initially, attempts were made to complement the *icd* mutant in *trans* by cloning *icd* and its native promoter into the pSUP203 vector; however, all pSUP203-*icd* vectors isolated from *E. coli* SM10 contained single nucleotide substitutions that resulted in amino acid changes in *icd* that inactivated isocitrate dehydrogenase (data not shown). Since the *icd* mutant is unable to grow on 3M + glucose, *icd* mutants that spontaneously resolved the merodiploid were isolated on 3M + glucose agar plates as described in the Methods. The reversion rate of the *icd* mutant to a wild type phenotype grown in LB20 overnight was calculated to be 1 out of 1.6 × 10^10^ cells. Additional file 3: Figure S3 shows the typical growth curves for M93Sm, the *icd* mutant and the restored *icd* strain in LB20 and 3M + 0.15% glucose. M93Sm and the restored *icd* strain were able to grow in 3M + 0.15% glucose unlike the *icd* mutant (Additional file 3: Figure S3A). Additionally, when the strains were grown in LB20 the final cell density returned to wild type levels when *icd* was restored (Additional file 3: Figure S3B). To determine if restoring *icd* restores pathogenicity, juvenile rainbow trout were challenged via immersion with M93Sm, the *icd* mutant and the restored *icd* strain at a dose of between 4 × 10^6^ and 8 × 10^6^ CFU/ml. After day 8, 26% (5/19) of the M93Sm challenged fish, 40% (6/15) of the restored *icd* challenged fish and 95% (19/20) of the *icd* mutant challenged fish survived (Fig. 8). There was no statistically significant difference between M93Sm and the restored *icd* strain ( *p*= 0.50). Again, there was a statistically significant difference between M93Sm and the *icd* mutant (*p* < 0.00004). The results indicate that when the merodiploid present in the *icd* mutant is resolved, wild type levels of growth in 3M + 0.15% glucose and LB20 and pathogenicity against juvenile rainbow trout is returned.

## Discussion

The tricarboxylic acid (TCA) cycle is involved in the generation of energy through the oxidation of acetate. TCA intermediates serve as precursor metabolites for the synthesis of amino acids and peptidoglycan. The M93Sm genome along with the published genomes of *V. anguillarum* strains 775, 96F, M3, NB10, RV22, 90-11-286 were examined for TCA cycle enzymes and the following genes were found: *gltA*, *acnB, icd, sucAB, sucCD, sdhCDAB, frdABCD, fumA,* and *mdh* (Fig. 1 and Table 3). Additionally, *cra*, which encodes the repressor of fructose metabolism in *S. enterica* and *E. coli* and has previously been shown to be essential for *S. enterica* virulence, is present in the *V. anguillarum* genomes [22].

When in a nutrient limited environment, bacteria must be able to synthesize any essential metabolites that are not freely available in order to grow. Previous studies have shown that mutations in central metabolism genes result in attenuation of virulence in several intracellular pathogens including *S. enterica,* uropathogenic *E. coli* (UPEC), *M. tuberculosis* and *E. ictaluri* [17–19, 21, 23–26]. These observations suggest that central metabolism is necessary for these intracellular pathogens to function inside the nutrient-limited environment of the phagosome; however, *V. anguillarum* is not an intracellular pathogen. While some studies have suggested that *V. anguillarum* can survive internally in fish epithelial cells and CHSE cells (derived from pooled embryonic cells from *Oncorhynchus tshawytscha*), more recent studies have demonstrated that *V. anguillarum* actively evades phagocytosis by fish epithelial cells and cannot survive for 24 h in macrophages [35–38]. In this study, fish were infected with *V. anguillarum* strains by either of two methods: intraperitoneal injection or immersion with both methods resulting in a similar percent survival when fish were challenged with M93Sm (20% for injection, see Fig. 2a and b; 0%~30% for immersion, see Fig. 3). Only the *icd* mutant had a statistically significant higher level of survival compared to the wild type, 100% for IP injection (Fig. 2a and b) and 90% for immersion (Fig. 3). It is not thought that reversion of the merodiploid to a wild type phenotype caused the other metabolism mutants to be virulent because chloramphenicol resistant colonies were isolated from the organs of dead fish. IP injection bypasses the need for invasion. No mortalities resulted from IP injection with the *icd* mutant indicating that *icd* is required for *V. anguillarum* persistence and growth in fish tissues. Rainbow trout infected with the *icd* mutant via immersion and subsequently challenged with the M93Sm wild type showed 100% survival (Fig. 4) demonstrating that the *icd* mutant had immunoprotective effects and elicited an adaptive immune response. Moreover, as a proof of concept, the data suggest that an *icd* deletion mutant could be the basis for a live attenuated vaccine against *V. anguillarum* infection.

Our observation that a knockout of the *icd* gene results in attenuation of virulence raises the question of whether expression of required virulence genes is significantly reduced in the mutant and, therefore, results in attenuation. We previously identified and characterized three hemolysin/cytolysin genes and their encoded proteins secreted by *V. anguillarum*: *plp*, *vah1* and *rtxA* [7, 9, 11]. While mutations in *plp* and *vah1* have modest effects on virulence against fish epithelial cells and fish, a knockout mutation in *rtxA* is avirulent in fish [7, 9, 11]. All metabolism mutants exhibited no significant declines in the expression of three hemolysins (Fig. 5) and most of the mutants exhibited increased expression. Accordingly, the *icd* mutant is not attenuated by the lack of hemolysin production because the decrease in *rtxA* and *vah1* expression was not significant; however, future studies could examine the expression of other virulence factors. It is unclear why expression of *plp* and *vah1* is increased in the metabolism mutants. Minato et al. [39] demonstrated the accumulation of acetyl-CoA in *Vibrio cholerae* central metabolism mutants resulted in an increased expression of its virulence gene activator ToxT. It is possible that accumulation or depletion of certain metabolites in *V. anguillarum* could increase hemolysin/cytolysin expression. Expression of *hlyU*, the positive regulator of the both the *vah1 plp* gene cluster and the *rtxA* gene cluster, was examined and shown to be up-regulated in the *sucA* and *mdh* mutants (data not shown) [40]. However, the increased expression of *hlyU* may not be the sole explanation for the increased expression of *plp* and *vah1* because an increase in expression of *rtxA* should have also occurred.

The growth rate and final cell density was determined for all metabolism mutants grown in LB20 for 24 h. The slowest growing mutant, *fumA,* was as virulent as the wild type while the mutant with the lowest final cell density, *icd*, was attenuated suggesting that decreased final cell density results in a loss of pathogenicity against rainbow trout (Figs. 2, 6, Tables 4 and 5). When the mutation in *icd* was resolved, the restored *icd* strain demonstrated the wild type phenotype for both growth and pathogenicity (Additional file 3: Figure S3A and B and Fig. 8). While it is possible that the insertional mutation affected the expression of the two genes flanking *icd* (ribosomal large subunit pseudouridine synthase E (Accession number: WP_017043910.1) and cold shock domain protein CspD (Accession number: WP_013857087.1)), it is unlikely as neither gene is part of an operon that includes *icd*. Since isocitrate dehydrogenase catalyzes the formation of α-ketoglutarate (the immediate precursor of glutamate) from isocitrate, the *icd* mutant is auxotrophic for glutamate (Fig. 7a). Our data demonstrate that the *icd* mutant stops growing once exogenous glutamate or its derivatives are exhausted (Fig. 7b and c). The data also demonstrate the decreased growth yield was not do to a reduction of ATP production as addition of gluconate or succinate did not restore growth to a wild type cell density. It is interesting that the only other auxotrophic mutant, *sucA*, grows to a wild type cell density in LB20 and is as virulent as the wild type considering it cannot synthesize succinyl-CoA, a metabolite needed for the synthesis of lysine, methionine and diaminopimelic acid. Presumably, succinyl-CoA or its derivatives are not limiting in LB20 or in fish tissues. Furthermore, this also suggests that the *icd* mutant is primarily starved for glutamate and would not need to synthesize succinyl-CoA by metabolizing glutamate to α-ketoglutarate. We hypothesize that during infection the *icd* mutant is unable to obtain enough α-ketoglutarate derivatives to grow to a wild type cell density and, therefore, cannot reach a cell density necessary for a successful systemic infection. In support, it has previously been demonstrated that a *V. anguillarum* M93Sm *mugA* mutant that was unable to grow in salmon intestinal mucus was avirulent against Atlantic salmon [41]. Additionally, when *V. anguillarum* 775 was cured of its plasmid-encoded siderophore, the mutant was unable to sequester iron and exhibited decreased virulence [42, 43].

M93Sm is an O2α serotype and the presumed infection route is through the gastrointestinal tract as no necrotic skin lesions have ever been observed with this strain (unpublished data). The in vitro growth experiment (Table 5) suggests that there are not enough α-ketoglutarate derivatives in intestinal mucus to support the growth of the *icd* mutant to a wild type cell density even though it is the metabolite with the second highest concentration (3.03 mM) in rainbow trout mucus [44]. It should be noted that for in vitro growth experiments the concentrations of glutamate and glutamine in the mucus are not known and the growth conditions represent an ideal environment for growth; *V. anguillarum* does not have to evade the fish immune system or compete with commensal bacteria and it is not expected that the *icd* mutant will grow to the cell density shown in the in vitro growth experiments in the fish. As demonstrated by Muroga et al., [45] *V. anguillarum* found in the spleen and intestine of moribund fish challenged via immersion only reached a cell density of 4.0 × 10^8^ CFU/g and 2.5 × 10^7^ CFU/g respectively. Altinok et al. [46] showed a *V. anguillarum* succinate dehydrogenase mutant was avirulent against rainbow trout when injected at a dose at 10^5^ CFU. Similar to our results, the authors showed that the succinate dehydrogenase mutant grew to a cell density slightly lower than the wild type at 12 h; however, the authors failed to show the growth yield at 24 h. Further, the authors did not create a complement strain to demonstrate that the loss of virulence was solely do to mutating *sdhB*. Most importantly, the ATCC has redesignated their strain as a *Pseudomonas* species.

## Conclusions

Seven *V. anguillarum* metabolism mutants were created and examined for pathogenicity against juvenile rainbow trout, hemolysin/cytolysin expression and growth in rich media. Of the central metabolism mutants, only the *icd* mutant showed strong attenuation in virulence, which did not result from a decrease in virulence factor expression. In addition, only the *icd* mutant had a final cell density that was lower than the wild type, which resulted from the inability to synthesize α-ketoglutarate and downstream metabolites. Taken together, the data suggest that during infection, if *V. anguillarum* is unable to synthesize essential molecules (e.g. α-ketoglutarate/2-oxoglutarate) and when those molecules or their derivatives (e.g. glutamate, glutamine) become limiting in the host, *V. anguillarum* will be unable to grow to a density necessary to sustain a systemic infection of the host.

## Additional files

Additional file 1: Figure S1. Hemolytic activity of various *V. anguillarum* strains grown on fish blood agar. Colonies grown overnight on LB20 plates were tooth picked onto LB20 + 5% trout blood agar plates. The diameter of the zones of hemolysis were measured after 7 h and 23 h of growth at 27 °C. Between marked strains and M93Sm: * *p* < 0.05 and ** *p* < 0.01. Error bars represent 1 standard deviation (PDF 146 kb)

Additional file 2: Figure S2. Final cell densities (OD_600_) of *V. anguillarum* WT (M93Sm) and the *icd* mutant after 24 h of growth in LB20 supplemented with or without 118 mM glucose and 118 mM succinate. Error bars represent 1 standard deviation (PDF 145 kb)

Additional file 3: Figure S3. Growth curves of *V. anguillarum* strains M93Sm (WT), the *icd* mutant and the restored *icd* strain grown in **A)** 3 M + 0.15% glucose and **B)** LB20. In each experiment cells grown overnight in LB20 at 27 °C were washed in NSS and used to inoculate the appropriate media. Cultures were incubated at 27 °C in a shaking water bath (200 rpm) and at various times after inoculation, samples were taken for determination of optical density at 600 nm (OD_600_). Different letters indicate statistical significance among groups (*p* < 0.05). Statistical analysis was based on data of stationary phase cultures (>12 h) (PDF 142 kb)

## Abbreviations

3M: Marine Minimum Median
*acnB*: Aconitate hydratase B
ANOVA: Analysis of variance
ATCC: American Type Culture Collection
ATP: Adenosine triphosphate
CFU: Colony Forming Unit
CHSE: Chinook salmon embryo
Cm: Chloramphenicol
*cra*: functional fructose repressor
CspD: Cold Shock Domain Protein
DNA: Deoxyribonucleic acid
*frdABCD*: Fumarate reductase (flavoprotein subunit, iron-sulfur subunit, anchor subunit, anchor subunit)
*fumAC*: Aerobic fumarate hydratase (class I, class II)
*fumB*: Anaerobic fumarate hydratase (class I)
*gltA*: Type II citrate synthase
HlyA: Hemolysin A
HlyU: Positive transcriptional regulator of the *plp*/*vah1* and the *rtx* gene clusters
H-NS: Histone-like Nucleoid-structuring
IACUC: The Institutional Animal Care and Use Committee
*icd*: isocitrate dehydrogenase
IP: intraperitoneal injection
Km: kanamycin
LB: Lysogeny broth
LB20: Lysogeny broth containing 2% NaCl
MARTX: Multifunctional Autoprocessing Repeats-in-Toxin
*mdh*: Malate dehydrogenase
*mugA*: mucus utilization gene A
NCBI: National Center for Biotechnology Information
NSS: Nine Salts Solution
NSSM: NSS supplemented with fish gastrointestinal mucus
PCR: Polymerase chain reaction
Plp: Phospholipase
qRT-PCR: Real-Time Quantitative Polymerase Chain Reaction
RAST: Rapid Annotation using Subsystem Technology
RNA: Ribonucleic Acid
RtxA: Repeat-in-toxin A
*sdhCDAB*: Succinate dehydrogenase (cytochrome b556 subunit, membrane anchor subunit, flavoprotein subunit, iron-sulfur protein)
Sm: Streptomycin
*sucAB*: 2-oxoglutarate dehydrogenase (E1 component, E2 component)
*sucCD*: Succinyl-CoA synthetase (beta subunit, alpha subunit)
TCA: Tricarboxylic acid
UPEC: uropathogenic *Escherichia coli*
Vah1: *Vibrio anguillarum* hemolysin 1
