# Supplementary figures and images for: Isocitrate dehydrogenase mutation in Vibrio anguillarum results in virulence attenuation and immunoprotection in rainbow trout (Oncorhynchus mykiss)
Source: BMC Microbiol. 2017 Nov 14;17:217. doi: 10.1186/s12866-017-1124-1 (PMC5686843; doi:10.1186/s12866-017-1124-1)

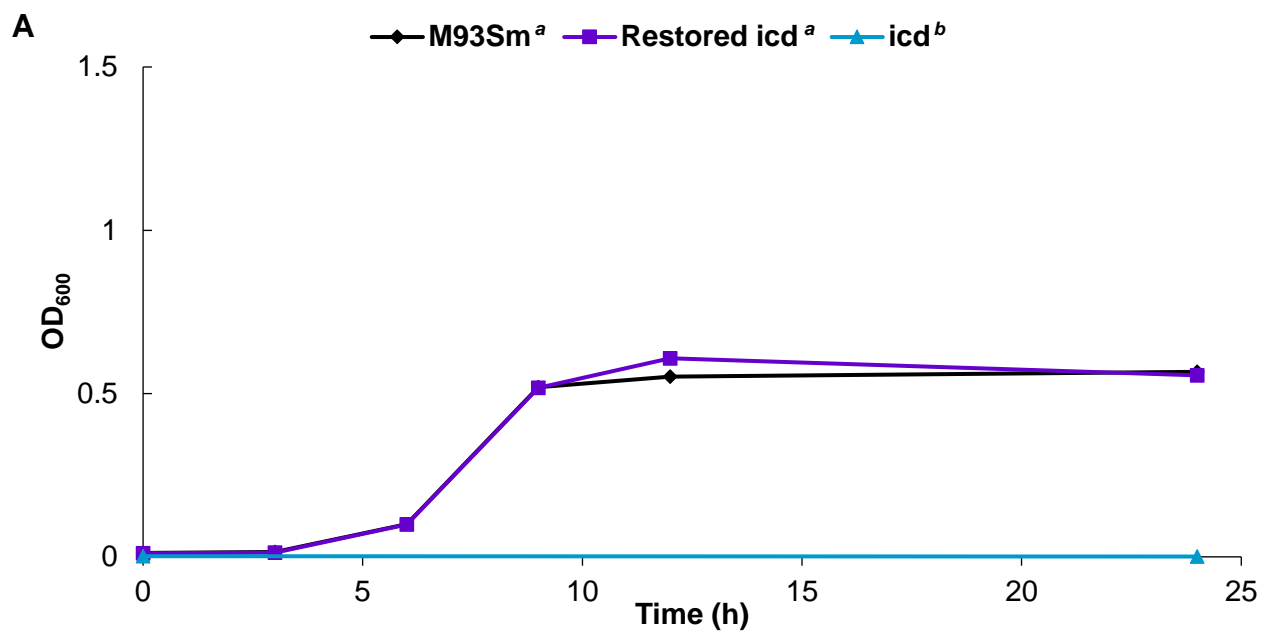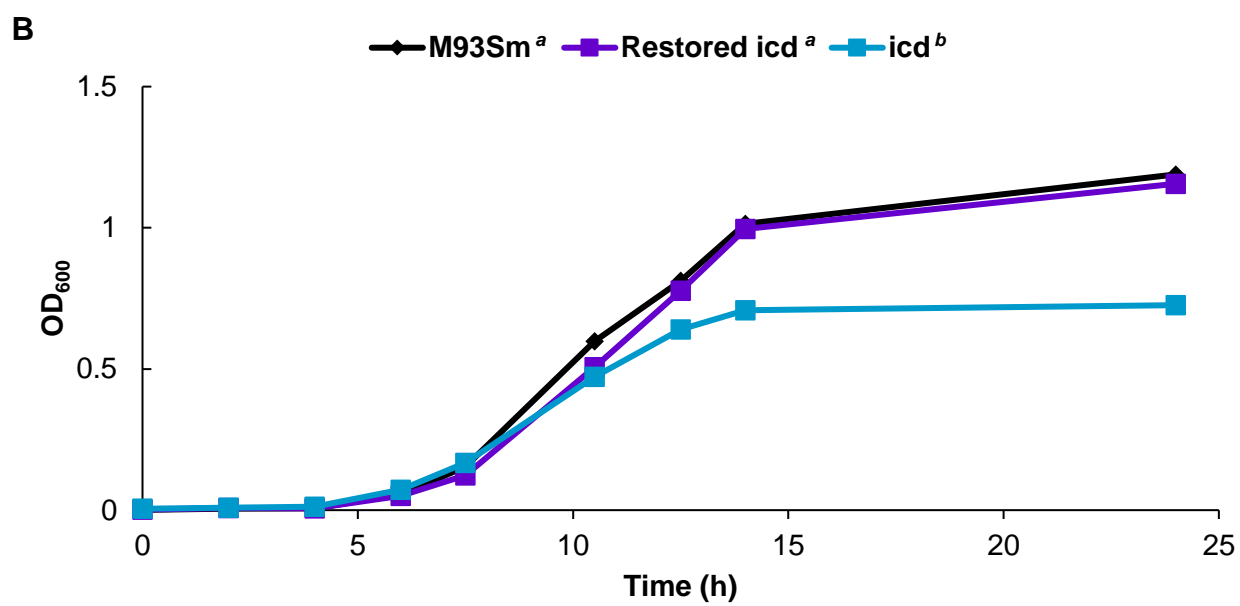

Supplement: Supplementary file 1 — Hemolytic activity of various V. anguillarum strains grown on fish blood agar. Colonies grown overnight on LB20 plates were tooth picked onto LB20 + 5% trout blood agar plates. The diameter of the zones of hemolysis were measured after 7 h and 23 h of growth at 27 °C. Between marked strains and M93Sm: * p < 0.05 and ** p < 0.01. Error bars represent 1 standard deviation (PDF 146 kb) [file 12866_2017_1124_MOESM1_ESM.pdf]

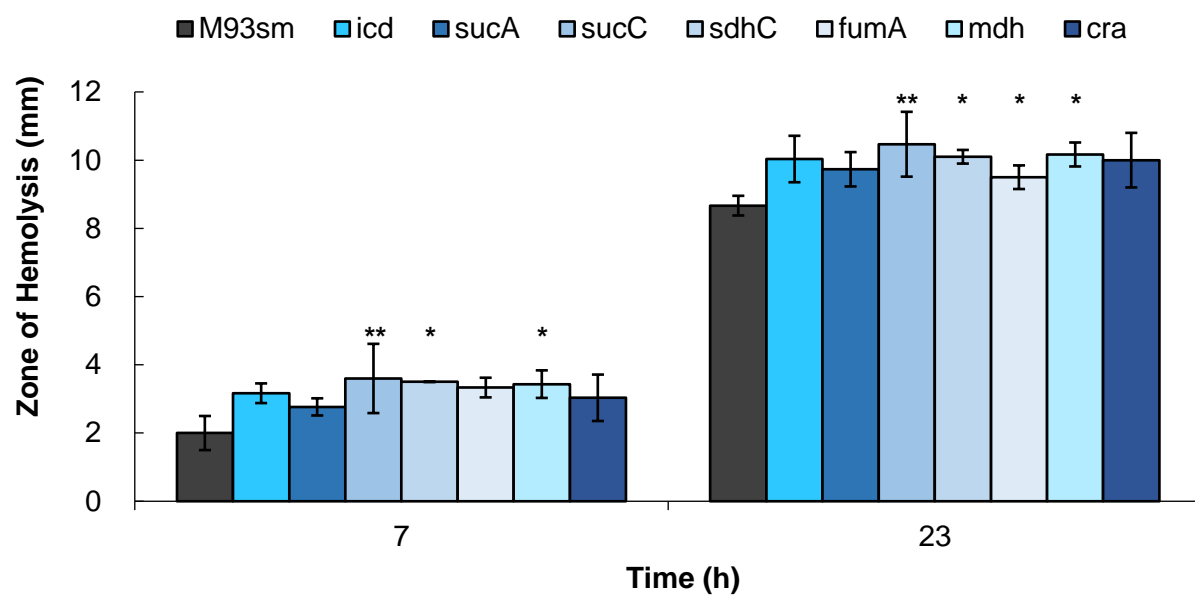

Supplement: Supplementary file 2 — Final cell densities (OD600) of V. anguillarum WT (M93Sm) and the icd mutant after 24 h of growth in LB20 supplemented with or without 118 mM glucose and 118 mM succinate. Error bars represent 1 standard deviation (PDF 145 kb) [file 12866_2017_1124_MOESM2_ESM.pdf]

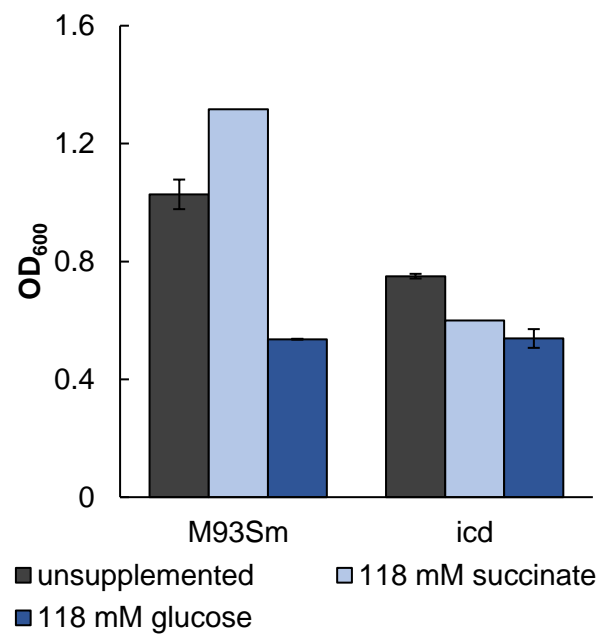

Supplement: Supplementary file 3 — Growth curves of V. anguillarum strains M93Sm (WT), the icd mutant and the restored icd strain grown in A) 3 M + 0.15% glucose and B) LB20. In each experiment cells grown overnight in LB20 at 27 °C were washed in NSS and used to inoculate the appropriate media. Cultures were incubated at 27 °C in a shaking water bath (200 rpm) and at various times after inoculation, samples were taken for determination of optical density at 600 nm (OD600). Different letters indicate statistical significance among groups (p < 0.05). Statistical analysis was based on data of stationary phase cultures (>12 h) (PDF 142 kb) [file 12866_2017_1124_MOESM3_ESM.pdf]
